# The Reliability and Validity of an Instrumented Device for Tracking the Shoulder Range of Motion

**DOI:** 10.3390/s25123818

**Published:** 2025-06-18

**Authors:** Rachel E. Roos, Jennifer Lambiase, Michelle Riffitts, Leslie Scholle, Simran Kulkarni, Connor L. Luck, Dharma Parmanto, Vayu Putraadinatha, Made D. Yoga, Stephany N. Lang, Erica Tatko, Jim Grant, Jennifer I. Oakley, Ashley Disantis, Andi Saptono, Bambang Parmanto, Adam Popchak, Michael P. McClincy, Kevin M. Bell

**Affiliations:** 1 Department of Bioengineering, University of Pittsburgh, Pittsburgh, PA 15260, USA; rar189@pitt.edu (R.E.R.); mir67@pitt.edu (M.R.); leg102@pitt.edu (L.S.); sik79@pitt.edu (S.K.); 2 ēlizur, Pittsburgh, PA 15237, USA; jenniferlambiase@gmail.com (J.L.); stephanylang@elizur.com (S.N.L.); ericatatko@elizur.com (E.T.); jimgrant@elizur.com (J.G.); 3 Department of Orthopaedic Surgery, University of Pittsburgh, Pittsburgh, PA 15213, USA; luckcl@upmc.edu (C.L.L.); oakleyji@upmc.edu (J.I.O.); mcclincymp@upmc.edu (M.P.M.); 4 Department of Health Information Management, University of Pittsburgh, Pittsburgh, PA 15260, USA; dhp68@pitt.edu (D.P.); vayu.putraadinatha@pitt.edu (V.P.); made.yoga@pitt.edu (M.D.Y.); andi.saptono@pitt.edu (A.S.); parmanto@pitt.edu (B.P.); 5 Department of Physical Therapy, Duquesne University, Pittsburgh, PA 15282, USA; disantisae@chp.edu; 6 Department of Physical Therapy, University of Pittsburgh, Pittsburgh, PA 15219, USA; ajp64@pitt.edu

**Keywords:** rotator cuff repair, validity, reliability, inertial measurement unit

## Abstract

Rotator cuff tears are common in individuals over 40, and physical therapy is often prescribed post-surgery. However, access can be limited by cost, convenience, and insurance coverage. CuffLink is a telehealth rehabilitation system that integrates the Strengthening and Stabilization System mechanical exerciser with the interACTION mobile health platform. The system includes a triple-axis accelerometer (LSM6DSOX + LIS3MDL FeatherWing), a rotary encoder, a VL530X time-of-flight sensor, and two wearable BioMech Health IMUs to capture upper-limb motion. CuffLink is designed to facilitate controlled, home-based exercise while enabling clinicians to remotely monitor joint function. Concurrent validity and test–retest reliability were used to assess device accuracy and repeatability. The results showed moderate to good validity for shoulder rotation (ICC = 0.81), device rotation (ICC = 0.94), and linear tracking (from zero: ICC = 0.75 and RMSE = 2.41; from start: ICC = 0.88 and RMSE = 2.02) and good reliability (e.g., RMSEs as low as 1.66 cm), with greater consistency in linear tracking compared to angular measures. Shoulder rotation and abduction exhibited higher variability in both validity and reliability measures. Future improvements will focus on manufacturability, signal stability, and force sensing. CuffLink supports accessible, data-driven rehabilitation and holds promise for advancing digital health in orthopedic recovery.

## 1. Introduction

Rotator cuff tear is a common condition, affecting approximately 6% to 22% of individuals over the age of 40, with incidence increasing with age and successful treatment significantly improving quality of life [1,2]. Physical therapy (PT) is a cornerstone of recovery following rotator cuff repair (RCR) surgery, as it reduces stiffness and increases range of motion (ROM) through prescribed exercises [3,4,5]. However, postoperative PT for RCR patients can cost up to USD 3000 over a six-month period, with insurance status influencing access to care [6,7]. Barriers such as cost and convenience often hinder treatment adherence, with as low as 32% of patients receiving PT within a year of surgery [8]. Non-compliance to the rehabilitation protocol, defined as incomplete or incorrect execution of prescribed treatments, can result in healthcare inefficiencies, prolonged symptoms, extended therapy durations, decreased patient well-being, hospital readmissions, and additional surgical interventions [9,10,11].

Home-based therapy, supported by telemedicine in other medical fields, has reduced barriers to compliance and lowered medical costs [12,13,14,15,16]. However, effective, scalable monitoring of prescribed exercises remains limited due to the lack of portable, precise, interactive, and cost-effective tools [13,17,18,19]. While simplified IMU systems and wearable devices have been explored for joint motion tracking, they often suffer from limitations in spatial resolution, calibration reliability, and integration into the home-based environment [20]. Previous studies have demonstrated the value of IMU-based systems for the supervised assessment of shoulder ROM during rehabilitation, particularly in clinical or athletic settings. For example, Tranquilli et al. [21] used an IMU to assess joint function in an injured athlete, highlighting the potential for remote clinical monitoring. Similarly, Parel et al. [22] implemented an IMU-integrated rehabilitation protocol that provided real-time feedback and improved patient engagement during the recovery process. These studies emphasize the feasibility and benefit of wearable IMUs in supervised rehabilitation contexts.

To address the challenges of these systems and expand their applicability to home-based environments, the CuffLink system has been developed to aid patients after unilateral rotator cuff repair surgery. CuffLink is a joint function monitoring system consisting of a mechanical motion-tracking device (Strengthening and Stabilization System, ēlizur, Pittsburgh, PA, USA) [23] that enables controlled and protected ROM exercises to be completed at home, along with a web-based software application (interACTION, University of Pittsburgh, Pittsburgh, PA, USA) [24,25,26] for managing and communicating joint function data. Sensors integrated into the Strengthening and Stabilization System (SSS), including inertial measurement units (IMUs), collect joint function data, which are stored on a smartphone or tablet and streamed to the clinician portal. The system incorporates BioMech Health IMUs (Richmond, VA, USA), which stream three-dimensional motion data in real time using a wireless, self-calibrating platform. These IMUs were placed above the elbow and wrist of the participant to track the shoulder range of motion. Unlike many low-complexity IMU systems, BioMech sensors are capable of capturing multi-segment kinematics without manual recalibration. Their plug-and-play design provides an affordable, efficient, and reliable method for monitoring joint motion outside clinical settings [27,28], enabling longitudinal assessment and therapist-guided remote monitoring. The sensors embedded in the SSS device and positioned externally enable the robust tracking of both device movement and shoulder ROM.

CuffLink is intended to allow insurance-allocated visits to be shifted to later-phase rehabilitation, where in-clinic care is crucial for restoring activities of daily living. We hypothesize that this time shift in rehabilitation enabled by CuffLink will lead to reduced financial strain, a more standardized delivery of care, and improved functional recovery for RCR patients.

The goal of this study was to assess the concurrent validity and test–retest reliability of the CuffLink platform. This study aimed to validate the performance of CuffLink by having subjects, including asymptomatic controls and postoperative RCR participants, perform physical therapy exercises in a controlled laboratory setting. Optical motion capture, considered the gold standard for measuring kinematics, was used to compare the concurrent validity between the BioMech and CuffLink platforms within the asymptomatic and RCR groups. Differences between BioMech and CuffLink from two sessions of exercises completed by the asymptomatic group were used to assess test–retest reliability.

## 2. Materials and Methods

### 2.1. Participants

This study received Institutional Review Board approval from the University of Pittsburgh. Inclusion criteria for asymptomatic participants were as follows: individuals aged 18 years or older, with no history of shoulder injuries, and the ability to perform physical therapy exercises. For RCR participants, inclusion criteria were individuals aged 40–65, who were more than six weeks postoperative following a primary unilateral rotator cuff repair. Recruitment occurred through community announcements and referrals from participating UPMC orthopedic surgeons between April and July 2024. The in-person enrollment visit took place at the University of Pittsburgh Human Movement and Balance Laboratory (HMBL).

A minimum of fifteen participants was estimated to detect a moderate positive correlation (r = 0.7) with 90% power and a two-tailed significance level of *p* < 0.05. To account for potential dropout and to ensure robust validation of both the IMU-based and mechanical tracking systems, the sample size was inflated to eighteen per group. A total of thirty-six participants were recruited for this study: eighteen asymptomatic controls who completed two testing sessions and eighteen RCR participants who completed one session. Participants were compensated for participation. Sex and race were not predetermined criteria for recruitment; participants were enrolled consecutively based on their availability and eligibility, which led to the observed demographic distribution (Table 1) in each group.

### 2.2. Instrumentation

CuffLink is a motion-tracking platform integrated with interACTION, a companion mHealth system that was designed to work with the SSS mechanical exerciser. The SSS device, developed by ēlizur (Pittsburgh, PA, USA), enables at-home passive motion of the glenohumeral joint while also allowing for resistive protraction and retraction of the scapula without excessively loading the rotator cuff structures. The device is customizable to the individual’s anatomy, featuring adjustable height and handle settings. Participants can perform active, passive, and isometric exercises on the device. The SSS device was designed with both physical and software-based motion stops to keep participants within a safe motion window and automatically alert therapists if the limits were exceeded.

The interACTION app, developed by the University of Pittsburgh Health and Rehabilitation Informatics Lab (Pittsburgh, PA, USA), includes a mobile application for direct patient use and an online portal for clinician access. It receives data streamed from sensors placed along and inside the SSS. During testing, the interACTION app provided real-time ROM feedback for patients, and data were captured and saved in the portal for monitoring. BioMech Health IMUs, positioned above the elbow and near the wrist, tracked shoulder movement, which was then streamed to an iPad (Apple Inc., Cupertino, CA, USA). These systems together transform the instrumented SSS mechanical exerciser into a telerehabilitation system, enabling remote monitoring for clinicians and providing feedback for patients at home.

The sensors embedded in the SSS device include the triple-axis LSM6DSOX + LIS3MDL FeatherWing accelerometer, output range of ±90 degrees (Adafruit, Brooklyn, NY, USA); a two-phase incremental rotary encoder, output range of ±180 degrees (Taiss, Suzhou, China); and a VL530X time-of-flight sensor, output range of 30 mm to 620 mm (Adafruit, Brooklyn, NY, USA). The accelerometer tracked the device’s incline along the horizontal axis, the encoder measured internal/external rotation, and the time-of-flight sensor recorded forward and backward translation. All sensors were controlled by an Adafruit Feather Huzzah ESP8266 microcontroller (Brooklyn, NY, USA), equipped with Bluetooth capabilities. Data were transmitted via Bluetooth at a rate of 60 Hz, and the interACTION app was used for data capture and display.

**Figure 1:**
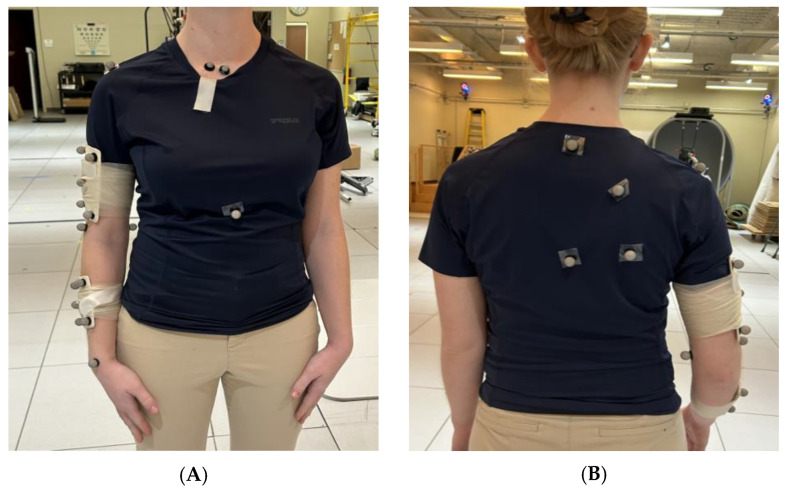

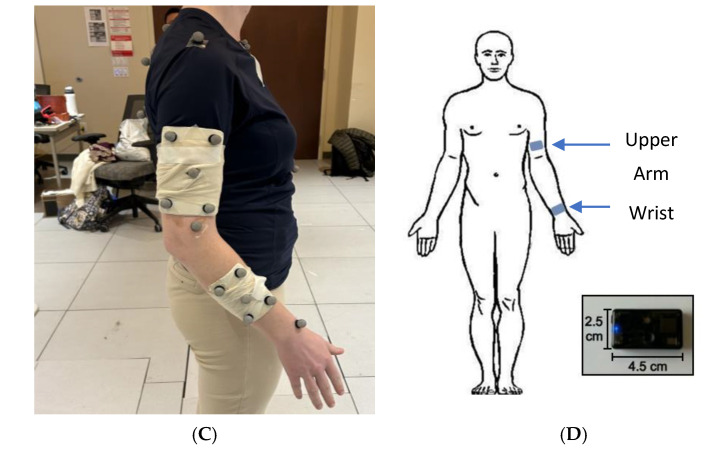
(**A**) The top left image is a frontal view of Vicon sensors placed on the chest and right arm. (**B**) The top right image is a posterior view of Vicon sensors placed on the back/right shoulder. (**C**) The bottom left image is a sagittal view of Vicon sensors placed on the arm. (**D**) The bottom right image is an illustration of the placement of the BioMech IMU sensors on the upper arm and wrist.

**Figure 2:**
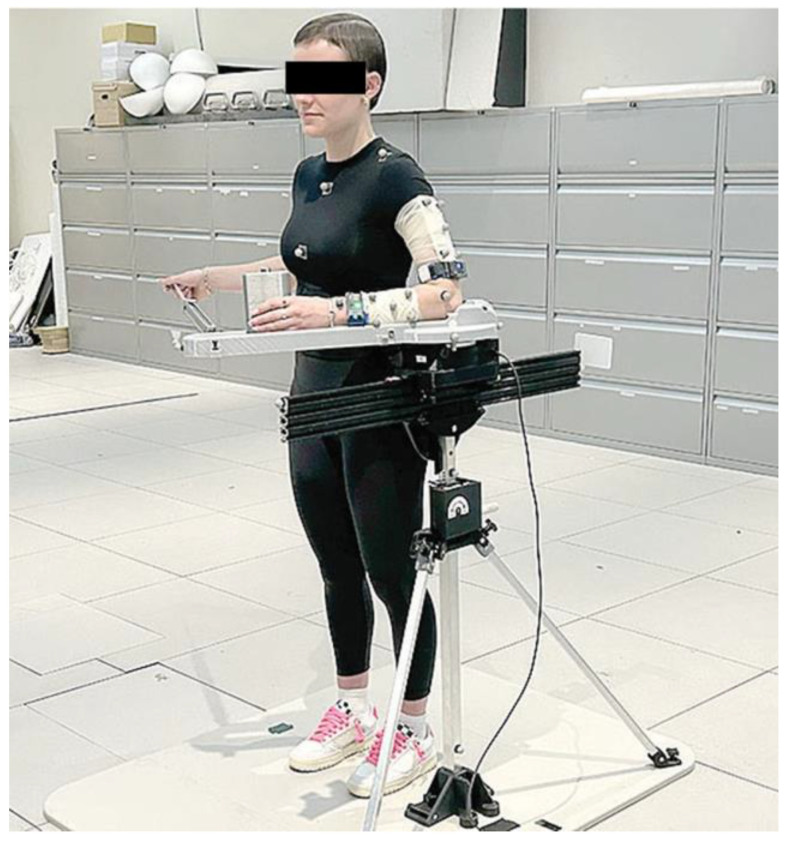
The participant completed internal rotation passive range of motion with Vicon sensors placed on the left side and BioMech sensors placed on the left wrist and above the elbow.

Measurements taken with the CuffLink system, and IMUs were corroborated using the Vicon optical motion capture system, which operates at 120 Hz with 14 T40S cameras (Oxford, UK). For both groups, 27 Vicon markers were used to track key anatomical and device movement (Figure 1A–C) [29,30,31]. Wearable sensors from BioMech Health were placed above the elbow and near the wrist of the participant to track shoulder movement (Figure 1D and Figure 2). Sensor and IMU placement were selected to align closely with bony anatomical landmarks and were applied by a trained clinical specialist to help minimize soft tissue artifacts (STAs).

### 2.3. Testing Procedures

**Figure 3:**
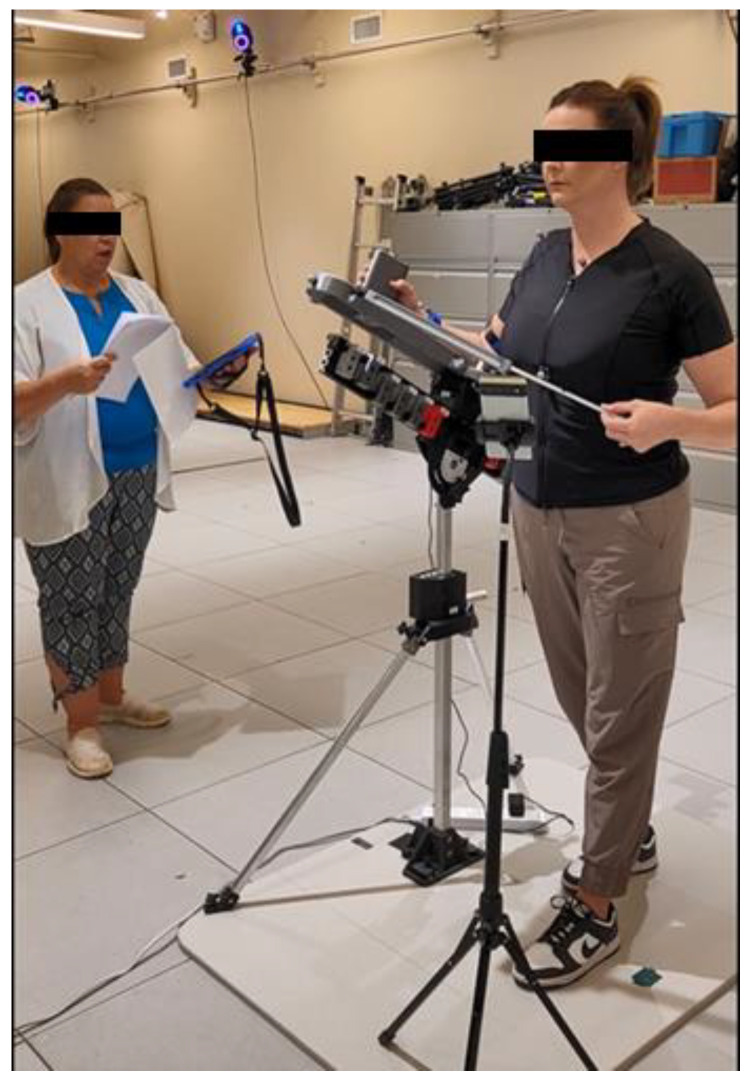
Participant performing forward elevation PROM while being verbally guided by a virtual clinical specialist.

Potential participants were screened over the phone prior to scheduling testing. Upon successfully passing the phone screening, an initial visit was scheduled at the HMBL. Prior to testing, a research coordinator obtained informed consent and conducted a set of screening questions as well as a demographic assessment. To standardize exercise execution, all participants were guided by a virtual clinical specialist who provided consistent verbal instructions for adjusting the device into position and performing the required movements. The participants logged into the interACTION app on the test phone and initiated the Bluetooth connection, and researchers started the Vicon recording. Participants were then verbally cued through the exercises by a researcher while using the interACTION app and SSS protocol and then completed the movements independently (Figure 3). Repetition count was standardized at either fifteen or thirty, depending on the exercise, and was monitored and counted by the researcher, with verbal reminders for progress and remaining repetitions. Participants were instructed to perform each movement at a self-selected, comfortable pace while maintaining proper form, including a stable torso. RCR participants were specifically instructed not to push through pain. To prevent injury or retearing of surgical repair, RCR participants performed only exercises appropriate for the six-week postoperative period, as determined by clinical specialists (SL and ET) and an orthopedic surgeon (MPM). Asymptomatic participants completed two sessions of the nine selected exercises, while RCR participants completed only one session and excluded the most advanced exercise, forward flexion active range of motion.

The nine exercises consisted of movements commonly performed during physical therapy after a rotator cuff repair:

1. Internal rotation passive range of motion (IR PROM);
2. External rotation passive range of motion (ER PROM);
3. Internal rotation active range of motion (IR AROM);
4. External rotation active range of motion (ER AROM);
5. Retraction active range of motion;
6. Protraction active range of motion;
7. Forward elevation passive range of motion (FE PROM);
8. Forward elevation active range of motion (FE AROM);
9. Abduction passive range of motion (ABD PROM).

Participants completed the following:

- Fifteen repetitions for IR PROM, ER PROM, IR AROM, ER AROM, and retraction/protraction AROM;
- Thirty repetitions for FE PROM, FE AROM, and ABD PROM.

Since the time-of-flight sensor was rigidly attached to one end of the rail, switching between arms caused a reversal in the magnitude of recorded distance and rotation values. To ensure consistency, IR/ER values were combined to standardize results across participants and sides.

To assess the concurrent validity of CuffLink, both asymptomatic and RCR participants performed exercises, while Vicon markers tracked key anatomical and device movements. CuffLink’s measured readings during these sessions were compared against Vicon motion capture system readings to determine the relationship between measurement error and the true value. Intraclass correlation coefficients (ICCs) and root mean square error (RMSE) values were used to evaluate measurement accuracy. The following metrics were analyzed for each movement: device rotation from zero and shoulder flexion during IR/ER; device displacement from zero during protraction/retraction; device displacement from the starting position and shoulder flexion during forward elevation; and device displacement from the starting position and shoulder abduction during abduction.

To assess test–retest reliability, the differences between CuffLink readings from the first and second testing sessions of asymptomatic participants were compared. ICCs and RMSE values were used to measure repeatability over time. The following metrics were analyzed for each movement: device rotation from zero and shoulder rotation during IR/ER exercises; device displacement from zero and shoulder flexion/extension during protraction/retraction; device displacement from the starting position and shoulder flexion during forward elevation; and device displacement from the starting position and shoulder abduction during abduction.

### 2.4. Data Processing

Raw data signals were converted into clinically relevant data streams. Vicon data underwent gap filling and labeling before being exported to Microsoft Excel (Version 2411) for subsequent analysis in MATLAB (Version R2022a, MathWorks Inc., Natick, MA, USA). Neither Vicon nor CuffLink data were filtered. BioMech IMU data were processed using a real-time proprietary algorithm; however, the specific postprocessing steps were not accessible. The interACTION and Vicon systems were not temporally synchronized, so comparisons were made using the peak value from each repetition.

To assess both validity and reliability, key movement parameters were extracted and processed for each repetition of each trial. Specifically, the maximum value of each repetition was identified for both rotational movements (degrees) and linear translations (centimeters). Extracted peak values from all valid repetitions were averaged to obtain a single session-level measurement for each participant and measurement system (CuffLink, BioMech, and Vicon). To ensure consistent performance and minimize edge effects, for exercises with fifteen reps, the first and fifteenth reps were excluded; for exercises with thirty repetitions, the first, second, twenty-ninth, and thirtieth repetitions were excluded. These exclusions were to account for potential inconsistencies due to participants’ adaptation to the task and to avoid edge effects related to protocol timing.

Although STAs can result from skin movement, muscle contractions, and inertial effects [32]—and are known to be task-, subject-, and segment-specific [33]—no additional filtering or corrections were applied to account for them. This decision was informed by the slow, controlled nature of the exercises, the relatively low dynamic loading, and the clinical placement of sensors directly over bony anatomical landmarks by a trained specialist. These factors were expected to minimize the influence of STA on peak movement measurements, which were the primary outcome of interest in this study.

### 2.5. Statistical Analysis

Statistical analyses were performed to assess concurrent validity (by comparing CuffLink and BioMech measurements to the gold-standard Vicon system for both asymptomatic and RCR participants) and test–retest reliability (by evaluating measurement consistency across two sessions for asymptomatic participants). Similar to other studies investigating the validity and reliability of IMU-based motion capture for upper-body biomechanics [34,35,36], three primary metrics were used: ICCs, RMSE, and Bland–Altman plots.

ICC values were calculated to assess inter-rater reliability and interpreted based on the following scale: poor (<0.5), moderate (0.50–0.75), good (0.75–0.90), and excellent (>0.90). The ICC significance level was set at α = 0.05, and 95% confidence intervals were reported. RMSE was used to quantify the average magnitude of error between measurement systems, while Bland–Altman plots visualized mean bias and 95% limits of agreement to assess systematic differences. Validity and reliability were benchmarked as ICC values between 0.75 and 1.00 (good to excellent agreement) and RMSE < 5 units. These complementary metrics provided a comprehensive assessment of agreement, accuracy, and measurement consistency. All statistical analyses were conducted using Microsoft Excel.

## 3. Results

### 3.1. Participants and Missing Data

Participant demographics are listed in Table 1. Eighteen asymptomatic participants provided consent and completed two sessions of the nine exercises. Eighteen RCR participants provided consent and completed one session of eight exercises.

Several partial/complete movements were excluded: Four tests recorded by CuffLink and one test recorded by BioMech with only one recorded repetition were excluded. Three participants were excluded from the validity analysis for forward elevation PROM and abduction PROM due to a missing acromion Vicon marker. One RCR participant had two device measurements excluded from validity comparisons due to a missing Vicon marker on the outer device. One control participant had their results excluded from reliability comparisons due to the incomplete protocol followed.

### 3.2. Concurrent Validity

ICC values for concurrent validity are displayed in Table 2, with bolded values meeting the benchmarks set for validity. Compared to Vicon, ICC values demonstrated concurrent validity to be excellent for one measurement, good for three, and poor for two (Table 2). The RMSE values of device distance measurements during protraction/retraction and combined forward elevation and abduction were within 5 cm between sessions, meeting the benchmark of <5; however, shoulder excursion and device rotation degrees were between 6 and 13 units.

**Figure 4:**
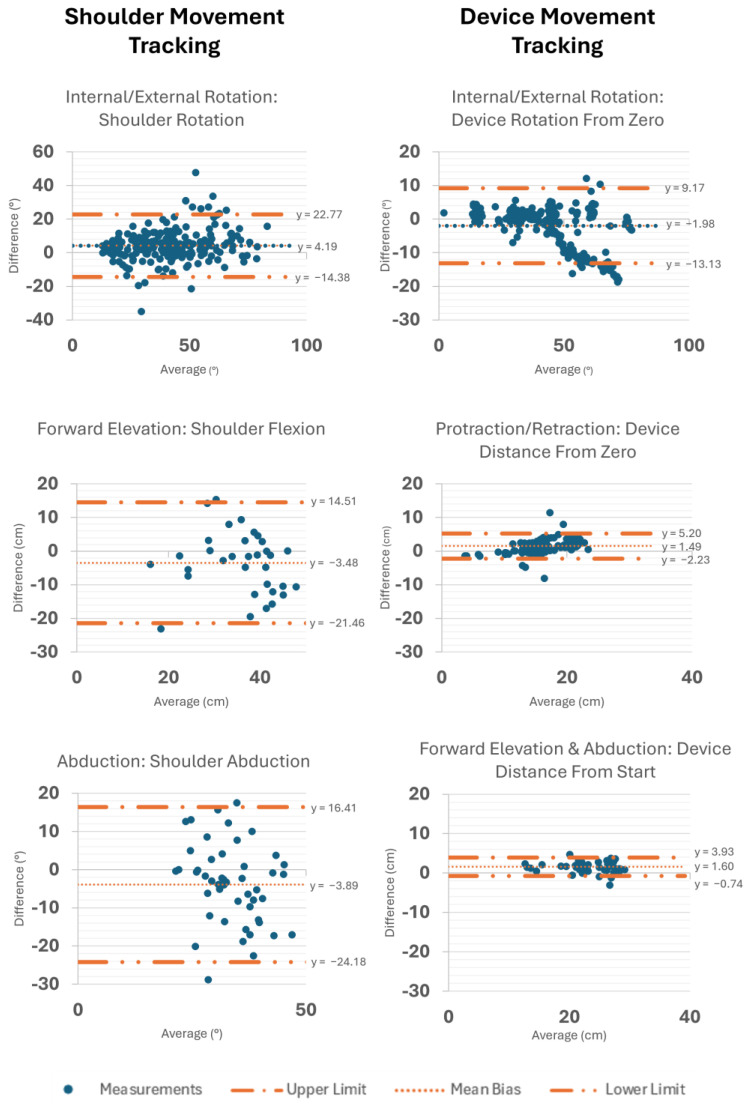
Bland–Altman plots to assess concurrent validity for BioMech (body) compared to Vicon during IR/ER, forward elevation, and abduction and for CuffLink (device) compared to Vicon during IR/ER, protraction/retraction, and combined forward elevation and abduction.

Bland–Altman plots were created to evaluate agreement between systems in both the RCR and asymptomatic groups (Figure 4). For shoulder rotation during IR/ER, the mean bias was 4.19°, with limits of agreement from −14.38 to 22.77°. For shoulder flexion during forward elevation, the mean bias was −3.48 cm, with limits of agreement from −21.46 to 14.51 cm. Shoulder excursion during abduction had a mean bias of −3.89°, with limits of agreement from −24.18 to 16.41°.

For device rotation during IR/ER, the mean bias was 1.98°, with limits of agreement from −13.13 to 9.17°. For device motion during protraction/retraction, the mean bias was 1.49 cm, with limits of agreement ranging from −2.23 to 5.20 cm. Finally, device motion during combined forward elevation and abduction showed a mean bias of 1.60 cm, and the limits of agreement were −0.74 to 3.93 cm. Across all movement types, most measurements fell within the limits of agreement, although some outliers were observed, particularly at higher average values.

### 3.3. Test–Retest Reliability

ICC values for test–retest reliability are displayed in Table 3, with bolded values in meeting the benchmarks set for reliability. Test–retest ICC values demonstrated the reliability between sessions of healthy control participants to be excellent for one measurement, good for two, moderate for two, and poor for one (Table 3). The RMSE of device distance measurements were within 5 cm between sessions, meeting the benchmark; however, shoulder excursion and device rotation degrees were between 5 and 10 units.

**Figure 5:**
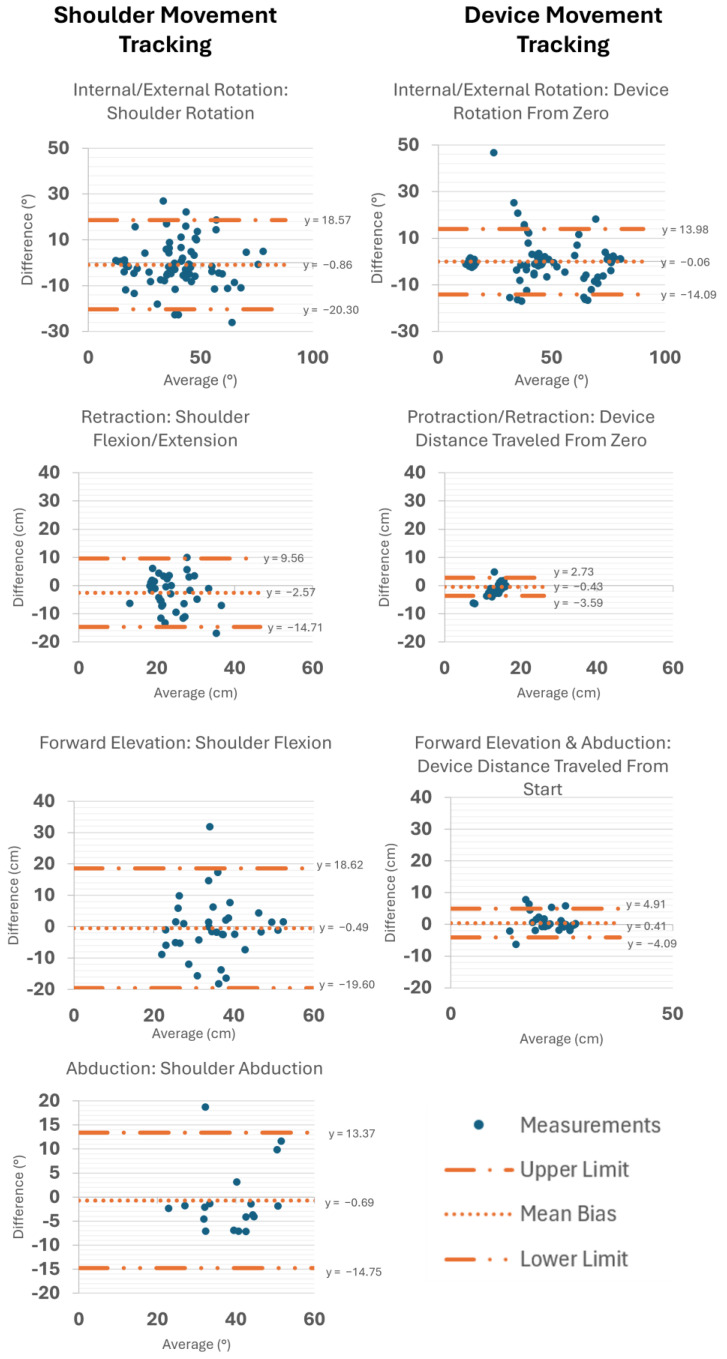
Bland–Altman plots to assess test–retest reliability for the shoulder (BioMech) during IR/ER, flexion/extension, forward elevation, and abduction and for the device (CuffLink) during IR/ER, protraction/retraction, and combined forward elevation and abduction.

Bland–Altman plots were created to evaluate the agreement between the first and second sessions across four primary movement types: IR/ER, retraction/protraction, forward elevation, and abduction (Figure 5). For shoulder rotation during IR/ER, the mean bias was −0.86°, with limits of agreement ranging from −20.30 to 18.57 degrees. Shoulder motion during retraction (flexion/extension) showed a mean bias of −2.57 cm, with limits of agreement from −14.71 to 9.56 cm. Forward elevation of the shoulder showed a mean bias of −0.49 cm, with limits of agreement from −19.60 to 18.62 cm. For shoulder motion during abduction, the mean bias was −0.69°, suggesting negligible systematic differences between sessions, with limits of agreement from −14.75 to 13.37°.

For device rotation during IR/ER, the mean bias was 0.06°, with limits of agreement from −14.09 to 13.98°. For device motion tracked during protraction/retraction, the mean bias was −0.43 cm, with limits of agreement ranging from −3.59 to 2.73 cm, indicating strong agreement between sessions, with minimal outliers. The device motion during combined forward elevation and abduction had a mean bias of 0.41 cm, with limits of agreement from −4.10 cm to 4.91 cm, demonstrating strong agreement and minimal session-to-session variability. Across all movements, the majority of measurements fell within the limits of agreement, supporting the repeatability of both shoulder and device-based measurements, though a few outliers were observed, particularly at higher ranges of motion.

## 4. Discussion

### 4.1. Concurrent Validity

The CuffLink system demonstrated good to excellent concurrent validity across multiple movement types when compared to the gold-standard Vicon system. Device-based measures, particularly for distance tracking during forward elevation and abduction, showed strong agreement and minimal bias, with ICC values as high as 0.94 and low RMSE values (as low as 2.02 cm and 6.02°). While rotation-based measures, such as IR/ER, showed slightly more variability, the overall bias remained small, and the proportional bias was modest. These results support the CuffLink system’s ability to accurately track a range of rehabilitation movements.

In contrast, shoulder-based measurements exhibited greater variability and lower agreement with Vicon, especially for angular movements. Forward elevation and abduction had the lowest ICCs (0.13 and 0.21, respectively). This variability may stem from participant movement differences, sensor repositioning, slight measurement drift, or the complexity of tracking multiplanar movements. A few larger outliers, particularly in forward elevation and abduction, may have also affected agreement and inflated RMSE values, which are sensitive to extreme data points. Despite this, the mean bias remained generally small, indicating that systematic errors were limited, even when variability was present.

Across exercises and joints, variability in agreement metrics tended to be higher for shoulder-based angular measurements (e.g., forward elevation and abduction) compared to device-based distance metrics. Shoulder flexion (forward elevation) and abduction showed the lowest ICC values and widest limits of agreement, suggesting that these complex, multiplanar movements may be less reliably tracked by the CuffLink system. In contrast, protraction/retraction and combined forward elevation/abduction exercises, particularly those captured via device distance, consistently demonstrated low RMSE values and high ICCs. These findings suggest that linear, device-referenced motions may be more suited for monitoring with the CuffLink system, while angular shoulder movements, especially in multiple planes, may be more prone to variability.

### 4.2. Test–Retest Reliability

The CuffLink system demonstrated strong test–retest reliability, particularly for distance-based metrics, with ICC values as high as 0.94 and consistently low RMSE values (as low as 1.66 cm and 5.41°). Low bias values across movements further support the device’s strong repeatability across a range of motion magnitudes. The narrow limits of agreement for most movements also highlight CuffLink’s stability for repeated use.

Shoulder-based measures, while showing good ICCs for some movements (e.g., IR/ER at 0.81), displayed greater session-to-session variability in angular tracking, particularly in forward elevation and abduction. This may suggest that these movements are more sensitive to session-to-session changes, potentially due to human variability, sensor positioning, or the challenges associated with tracking complex joint motion. Still, the lack of substantial proportional bias across shoulder movements suggests that errors did not systematically increase with greater motion ranges, supporting reliable relative performance.

Additionally, while more movements reached ICC benchmarks than RMSE benchmarks, this pattern suggests that the relative consistency between sessions was stronger than absolute agreement. This discrepancy may be explained by the presence of isolated outliers and reflects their impact on absolute agreement rather than indicating systemic reliability issues.

While the higher RMSE values observed in this study (10–13° for concurrent validity and 5–10° and 7 cm for test–retest reliability) may appear substantial at first, these values are consistent with those reported in prior IMU-based joint motion validation studies for upper-limb movements. Several studies noted that measurement errors in this range are not unexpected, particularly when measuring larger joint excursions or complex multiplanar shoulder movements. The reported RMSE values in the literature can range from 2 to 10° across different axes of rotation when comparing IMU to OMC systems during functional shoulder and upper-limb tasks, with authors concluding that such discrepancies may be acceptable depending on the application [35,37,38,39].

Despite these observed errors, most ICC and RMSE values remained within acceptable thresholds, supporting the CuffLink system’s potential for use in both clinical and home-based rehabilitation settings. Given the context of this study, where larger joint excursions are being evaluated rather than fine angular precision, the reported errors may not significantly impact clinical interpretation or patient safety during rehabilitation sessions. However, considering that these exercises are intended for individuals recovering from RCR surgery, further research is warranted to reduce errors and improve measurement precision. Overall, the error magnitudes reported in this study fall within a range commonly accepted in similar validation research and do not undermine the potential of the device for tracking broad movement trends during rehabilitation.

### 4.3. Limitations

Vicon cameras occasionally had difficulty detecting certain sensors due to anatomical features or clothing obstructing visibility. As a result, unresolved gaps during data processing necessitated the exclusion of some markers from the exported dataset. When key markers were missing, alternative markers and formulas were used to estimate the necessary data. These substitutions had a minimal impact on the results, as the estimated midpoints and coordinate systems were derived using anatomically consistent references and validated segment definitions. Additionally, the overall joint motion calculations rely on relative changes between body segments rather than absolute marker positions, further mitigating the influence of occasional marker loss on system or session-to-session agreement.

Bluetooth connectivity issues between the CuffLink system and the interACTION app also occurred multiple times during testing, highlighting the need for software updates to minimize delays and data gaps. Additionally, the sensors installed on the SSS have inherent limitations. Neither the instrumented device nor the wearable sensors can measure isometric force. While the current device can verify compliance with isometric exercises, integrating force sensors would enhance clinical insight. Furthermore, the wearable sensors track movement changes relative to the starting position, potentially leading to measurement offsets. For example, if a patient begins in three degrees of internal rotation, this initial offset is not captured in the reported measurements. This limitation is particularly relevant for exercises like retraction/protraction, where visual feedback (e.g., a mirror) is often necessary to ensure proper starting alignment.

Despite minimal systematic bias across all movements, the relatively wide limits of agreement and presence of outliers, especially in rotational and large-range movements, suggest areas for refinement. The higher variability observed in shoulder movements may be partially attributed to the limited access to BioMech sensor data, as only processed outputs were available. Without access to raw data or algorithmic details, it was not possible to fully assess or correct potential sources of error, such as offset deviations. Furthermore, as the BioMech sensors are self-calibrating, it is possible that internal processing or calibration algorithms introduced offsets that could not be verified or adjusted during postprocessing. Additionally, participants were instructed to perform the exercises to their comfort level, which may have contributed to differences between sessions.

While multiple sources of error could have contributed to measurement variability, such as marker misplacement, STAs, and sensor alignment challenges, the largest contributor is likely inadequate calibration of the BioMech and CuffLink systems. Shoulder movements such as abduction and forward elevation, which involve complex, multiplanar motion and are highly susceptible to STAs, consistently showed lower ICC values and wider limits of agreement. Although sensor misalignment and anatomical variability may have further affected angular accuracy, prior research suggests that STAs play a more dominant role [40]. These factors, combined with calibration limitations, likely contributed to the reduced agreement observed in angular shoulder measurements.

Future development efforts will focus on formalizing CuffLink calibration procedures and fine-tuning BioMech sensor protocols to reduce residual variability and improve the robustness of motion tracking. In parallel, planned enhancements to the CuffLink system include improvements in manufacturability, circuit design to reduce power loss, increased encoder resolution, and integration of a force sensor to better capture isometric exercise performance.

## 5. Conclusions

The CuffLink system demonstrated strong accuracy and repeatability across multiple shoulder movements, with high performance during distance-based tracking. Its consistency in both concurrent validity and test–retest reliability, along with minimal bias and acceptable variability, supports its use as a telehealth rehabilitation system within the clinical and home environments. While some variability was noted in angle-based shoulder measurements, likely due to anatomical complexity and external factors such as sensor placement or inadequate calibration, these findings provide valuable insights for continued refinement and optimization.

While CuffLink was designed primarily for home-based postoperative rehabilitation following rotator cuff repair, its architecture aligns with emerging applications of wearable sensors in supervised outpatient care, occupational ergonomics, and the assessment of motor disorders [41,42]. IMU systems, such as BioMech Health, offer real-time, wireless, self-calibrating solutions that are adaptable to both constrained and unconstrained environments. However, common barriers such as movement tracking accuracy and reliable data capture must be addressed before routine clinical adoption. By effectively addressing these barriers to remote rehabilitation, CuffLink has the potential to enhance postoperative care, support patient compliance, and drive the adoption of digital health technologies in orthopedic recovery and beyond. With further development, it may offer clinicians a powerful tool for personalized, data-driven treatment plans, improving outcomes and accessibility in musculoskeletal care.

## Figures and Tables

**Table 1:** Participant demographics.

Asymptomatic (N = 18)	
Age	34.3 (17.5)
Sex	12 Female, 6 Male
BMI	26.6 (6.5)
Race	83 (15) White, 11 (2) Black, 6 (1) Multiracial
Rotator cuff repair (N = 18)	
Age	55.3 (7.4)
Sex	8 Female, 10 Male
BMI	30.2 (7.0)
Race	89 (16) White, 11 (2) Black

Age and BMI are listed as mean (standard deviation). Race is listed as percent (number). N represents the total number of participants in each group.

**Table 2:** Concurrent validity ICC gold standard (1,1) and RMSE.

Exercise Name(s)	System	Measurement	Unit	Rating	ICC (95% CI)	N	RMSE	SD
IR/ER	BioMech	Shoulder rotation	deg	**Good**	0.81 (0.75–0.85)	204	10.34	±9.28
Forward Elevation	BioMech	Shoulder flexion	deg	Poor	0.13 (−0.09–0.33)	83	12.69	±11.76
Abduction	BioMech	Shoulder abduction	deg	Poor	0.21 (−0.07–0.47)	48	10.96	±10.25
IR/ER	CuffLink	Device rotation	deg	**Excellent**	0.94 (0.92–0.95)	412	6.02	±5.79
Protraction/Retraction	CuffLink	Device distance from zero	cm	**Good**	0.75 (0.68–0.85)	204	**2.41**	±1.89
Forward Elevation and Abduction	CuffLink	Device distance from start	cm	**Good**	0.88 (0.83–0.91)	132	**2.02**	±1.36

ICC values are shown with 95% confidence intervals. Bolded ICC categories (good to excellent) and RMSE values indicate agreement that meets validity benchmarks: ICC ≥ 0.75 and RMSE < 5 units. N represents the number of valid participant-level measurements included in the analysis for each movement.

**Table 3:** Test–retest reliability ICC test–retest (2,1) and RMSE.

Exercise Name(s)	System	Measurement	Unit	Rating	ICC (95% CI)	N	RMSE	SD
IR/ER	BioMech	Shoulder rotation	deg	**Good**	0.81 (0.71–0.88)	72	9.80	±9.74
Protraction/Retraction	BioMech	Shoulder flexion/extension	cm	Poor	0.44 (0.14–0.67)	35	6.62	±6.10
Forward Elevation	BioMech	Shoulder flexion	deg	Poor	0.47 (0.16–0.69)	35	9.62	±9.61
Abduction	BioMech	Shoulder abduction	deg	Moderate	0.69 (0.34–0.87)	18	7.00	±6.97
IR/ER	CuffLink	Device rotation	deg	**Excellent**	0.94 (0.91–0.95)	142	5.41	±5.35
Protraction/Retraction	CuffLink	Device distance from zero	cm	Moderate	0.57 (0.38–0.71)	70	**1.66**	±1.60
Forward Elevation and Abduction	CuffLink	Device distance from start	cm	**Good**	0.79 (0.66–0.88)	48	**2.31**	±2.27

ICC values are shown with 95% confidence intervals. Bolded ICC categories (good to excellent) and RMSE values indicate agreement that meets reliability benchmarks: ICC ≥ 0.75 and RMSE < 5 units. N represents the number of valid participant-level measurements included in the analysis for each movement.

## Data Availability

The generated datasets from this study are available upon request to the corresponding author.

## Abbreviations

The following abbreviations are used in this manuscript:

PT	Physical therapy
RCR	Rotator cuff repair
ROM	Range of motion
SSS	Strengthening and Stabilization System
IMU	Inertial measurement unit
STA	Soft tissue artifact
HMBL	Human Movement and Balance Laboratory
IR	Internal rotation
PROM	Passive range of motion
ER	External rotation
AROM	Active range of motion
ICC	Intraclass correlation coefficient
RMSE	Root mean square error
